# From neurodevelopment to neurodegeneration: the interaction of neurofibromin and valosin-containing protein/p97 in regulation of dendritic spine formation

**DOI:** 10.1186/1423-0127-19-33

**Published:** 2012-03-26

**Authors:** Yi-Ping Hsueh

**Affiliations:** 1 Institute of Molecular Biology, Academia Sinica, 128, Sec 2, Academia Rd, Taipei 11529, Taiwan

**Keywords:** Dendritic spine formation, IBMPFD, Neurodevelopmental disorder, Neurofibromatosis Type I, neurofibromin, statin, VCP/p97.

## Abstract

Both Neurofibromatosis type I (NF1) and inclusion body myopathy with Paget's disease of bone and frontotemporal dementia (IBMPFD) are autosomal dominant genetic disorders. These two diseases are fully penetrant but with high heterogeneity in phenotypes, suggesting the involvement of genetic modifiers in modulating patients' phenotypes. Although NF1 is recognized as a developmental disorder and IBMPFD is associated with degeneration of multiple tissues, a recent study discovered the direct protein interaction between neurofibromin, the protein product of the NF1 gene, and VCP/p97, encoded by the causative gene of IBMPFD. Both NF1 and VCP/p97 are critical for dendritic spine formation, which provides the cellular mechanism explaining the cognitive deficits and dementia found in patients. Moreover, disruption of the interaction between neurofibromin and VCP impairs dendritic spinogenesis. Neurofibromin likely influences multiple downstream pathways to control dendritic spinogenesis. One is to activate the protein kinase A pathway to initiate dendritic spine formation; another is to regulate the synaptic distribution of VCP and control the activity of VCP in dendritic spinogenesis. Since neurofibromin and VCP/p97 also regulate cell growth and bone metabolism, the understanding of neurofibromin and VCP/p97 in neurons may be applied to study of cancer and bone. Statin treatment rescues the spine defects caused by VCP deficiency, suggesting the potential role of statin in clinical treatment for these two diseases.

## Review

### Neurodevelopmental disorders and neurodegeneration

Neurons are highly differentiated cells composed of several specialized subcellular structures, including soma, dendrites, axon, and numerous synapses. All of these structures play specific roles in signal transduction between or within neurons. Besides dendrites and axons, dendritic spines are particularly interesting structures, which are the tiny protrusions (~0.5-1 μm in width and ~1-2 μm in length) extending from dendrites. The majority of excitatory synapses in the mammalian nervous system are localized at the tips of dendritic spines [1]. Morphology and density of dendritic spines are controlled by genetic program, neuronal activity, and environmental insults. Indeed, defects of neural development leading to alterations of neuronal morphology are associated with many neurological and neuropsychiatric disorders, including mental retardation, learning disability, autism, attention-deficient hyperactivity disorder, and schizophrenia [2,3]. On the other hand, impairment in maintenance of neuronal morphology is also frequently found in neurodegenerative disorders. For instance, synaptic loss has been suggested as a cause of dementia [4-6]. This review will focus on an example showing that neurodegeneration may be relevant with neurodevelopment in terms of regulation of neuronal morphology. It is regarding a recent finding of the interaction between neurofibromin and valosin-containing protein (VCP).

Neurofibromin, the protein product of the neurofibromatosis type I (*NF1*) gene, regulates the formation of dendritic spines [7], which explains at least partially why patients with NF1 suffer from cognitive defects. Recently, we further identified that VCP/p97 interacts with neurofibromin and demonstrated that VCP/p97 also controls dendritic spinogenesis of neurons [8]. Mutations in the VCP gene result in inclusion body myopathy with Paget's disease of bone and frontotemporal dementia (IBMPFD) and amyotrophic lateral sclerosis (ALS). The function of VCP in spinogenesis provides a potential explanation how VCP mutations result in dementia in patients. Although both neurofibromin and VCP/p97 are ubiquitously expressed in many tissues and are also involved in tumorigenesis [9-11] and bone metabolism [12,13], the current review focuses on the molecular functions of neurofibromin and VCP/p97 in neurons.

### Neurofibromatosis type I (NF1)

Neurofibromatosis type I (NF1, MIM:162200) is one of the most common autosomal dominant disorders affecting about one in 3,500 individuals. NF1 is characterized by skin pigmentations (café-au-lait spots and freckling), Lisch nodules in the iris, and formations of benign peripheral nerve sheath tumors (neurofibromas and schwannomas). In addition, many other features are frequently found in patients with NF1, including neurobehavioral developmental disorders as well as skeletal lesions and malformations. Children with NF1 are frequently associated with learning difficulty [14-16] and are more susceptible to autism and attention-deficient hyperactivity disorder [15-18]. Additionally, scoliosis, kyphosis, short stature, and tibial bowing are common skeleton manifestations occurring in NF1 patients [19]. Based on the symptoms identified in patients with NF1, it is clear that the NF1 gene plays important roles in cell growth (tumor formation), pigmentation, neuronal activity and function, and bone metabolism. Although NF1 is fully penetrant, the phenotypes of NF1 patients are extremely variable, even among patients in the same family. It is unclear why clinical features of NF1 patients vary so markedly. Genetic modifiers have been suggested to influence the impact of NF1 deficits [20,21].

### The function of neurofibromin in signaling

**Figure 1:**
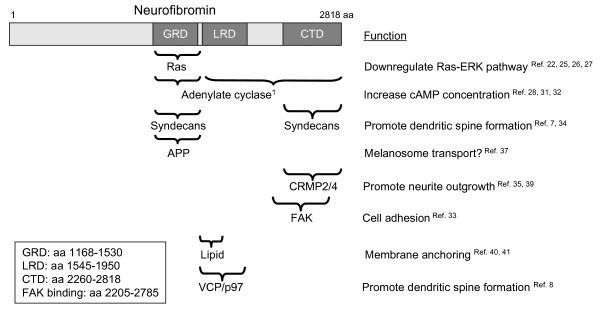
**The interacting proteins and corresponding functions of neurofibromin**. GRD, RasGAP-related domain; LRD, Leucine-rich domain; CTD, C-terminal domain. The amino acid (aa) residue number is based on the sequence of neurofibromin with type I GRD. The LRD can be further separated into the N-terminal Sec14-like domain (residue 1560-1699) for lipid binding and the C-terminal pleckstrin homology-like domain (residues 1713-1818). The braces indicate the interacting regions for each binding proteins. ^1^Note that the GRD and entire C-terminal region of neurofibromin are involved in activation of the adenylate cyclase. However, the mechanism is unknown. It is not clear whether the direct protein-protein interaction between neurofibromin and adenylate cyclase is involved.

The *NF1 *gene encodes a large protein of 2818 amino acid residues, named neurofibromin (Figure 1), with a well-known RasGAP-related domain (GRD, ~350 amino acid residues) at the central region of the protein [22]. The RasGAP activity is believed to account for the tumor suppression activity of neurofibromin [23-27], which is the most well known function of neurofibromin. In addition to downregulating the Ras pathway, neurofibromin increases adenylyl cyclase activity and thus upregulates the cAMP pathway through both G_αs_-dependent and G_αs_-independent pathways [28,29]. More details about the role of neurofibromin in signaling are available in the previous review [30].

### The obstacles of NF1 study

The NF1 gene was identified more than 20 years ago. However, several obstacles impede the progress of NF1 study. First of all, perhaps due to cytotoxic effects, it is not easy to express full length neurofibromin in the cell. Therefore it is difficult to perform genetic manipulation to study the molecular function of neurofibromin. Secondly, mutational hot spots are not present in the NF1 gene. The mutations identified from patients are dispersed throughout the entire NF1 gene. It is therefore difficult to figure out the pathogenic mechanism of NF1. Finally, with the exception of the GRD, neurofibromin lacks a recognizable enzymatic protein domain. Therefore, numerous efforts have been directed to identify neurofibromin-interacting proteins (Figure 1) [7,8,28,31-41]. Identification and characterization of proteins that interact with neurofibromin provide a better understanding of the molecular functions of neurofibromin.

### Neurofibromin regulates neuronal morphogenesis

The fact that patients with NF1 are frequently associated with multiple neurobehavioral developmental disorders suggests an important role of neurofibromin in brain development and function. Indeed, several studies have revealed the role of neurofibromin in neural development. Through its C-terminal domain, neurofibromin interacts with collapsing response mediator proteins, CRMP-2 and CRMP-4 [35,39]. This interaction regulates neurite outgrowth of neurons [39]. In addition to neurite outgrowth, our previous study showed that neurofibromin acts downstream of syndecan-2, a synaptic heparan sulfate proteoglycan, in the regulation of dendritic spine formation [7]. Neurofibromin interacts with syndecan-2 [34] and then activates the PKA-Ena/VASP pathway through the cAMP pathway to promote actin polymerization and bundle formation [7], thus promoting the formation of dendritic filopodia, the precursors of dendritic spines. The role of neurofibromin in dendritic spinogenesis explains at least partially why patients with NF1 are often associated with cognitive abnormalities. Moreover, although the PKA pathway is essential for dendritic filopodia and spine formation, activation of PKA alone is not sufficient for the process [7], possibly due to the involvement of multiple downstream pathways of neurofibromin in spinogenesis. Indeed, from a series of proteomic and biochemical studies, we identified that neurofibromin uses its leucine-rich domain (LRD) to directly interact with the valosin-containing protein (VCP) [8]. Knockdown of endogenous VCP reduces the density of dendritic spines, but does not noticeably influence their width or length. Interruption of the interaction between neurofibromin and VCP also reduces the spine density [8]. The studies suggest that neurofibromin regulates dendritic spine formation through at least two pathways: one cAMP-dependent and the other VCP-dependent.

### Valosin-containing protein (VCP) and degeneration disorders

IBMPFD MIM:167320, [42] is a dominant inherited disorder with three major symptoms: myopathy, osteolytic bone lesion, and frontotemporal dementia (FTD). Similar to NF1, IBMPFD is also fully penetrant, but expressivity is variable, suggesting the involvement of genetic modifiers in the influence of phenotype [43,44]. Around 80-90% and 50% of patients develop inclusion body myopathy and osteolytic lesions, respectively, with a mean age of 42 years. Only about 30-35% of patients had early-onset FTD typically presenting at age 53 years [42-44]. In addition to IBMPFD, recent human genetic analysis indicated that VCP mutations account for 1-2% of autosomal dominantly inherited amyotrophic lateral sclerosis (ALS) [45]. VCP interacts with the polyglutamine-containing aggregates that are found in patients with Huntington's and Machado-Joseph diseases [46]. VCP also associates with ubiquitinated protein aggregates and is involved in TDP-43-related frontotemporal dementia [47-49].

### Molecular functions of VCP

VCP, a multifunctional AAA (ATPases Associated with a variety of cellular Activities) protein, forms a homohexameric barrel and hydrolyses ATP to generate the mechanical force for its function as a molecular chaperon [50-52]. It possesses two ATPase domains (D1 and D2). The D2 domain carries the major ATPase activity, while the D1 domain is also the hexamerization domain of VCP. The N-terminal region (N-domain) of VCP is involved in its interaction with various adaptors. The identified IBMPFD mutations are highly clustered in the N- and D1 domains of VCP [42,53-55]. Therefore, IBMPFD mutations can change the conformation of the catalytic domains, alter the ATPase activity, and compromise the function of VCP [56]. Through the N-terminus interacting adaptors, VCP regulates a variety of cellular events, including cell-cycle control, membrane fusion, ubiquitin-dependent protein degradation, endoplasmic reticulum-associated protein degradation (ERAD), and autophagy [51,57-64].

So far, it is not clear how mutations in the VCP gene lead to IBMPFD or other degeneration diseases. An IBMPFD-associated VCP mutant was shown to induce aggregation of polyubiquitin-conjugated proteins in myoblastoma cells [65]. TDP-43-positive aggregates are also found in the muscles of patients with IBMPFD [48]. VCP mutations have also been shown to cause autophagy dysfunction, which may additionally contribute to the pathogenesis of IBMPFD [62,63,66]. Therefore, dysregulation of protein degradation has been proposed to play a role in IBMPFD and VCP-related disorders.

Although the formation of VCP-positive protein aggregates induced by IBMPFD mutation have been demonstrated in muscle, the significance of protein aggregation in neurons is not so clear. Expression of IBMPFD mutant VCP in cultured neurons reduces the density of dendritic spines, but does not induce VCP aggregation [8]. It suggests that VCP aggregation is not the cause of synaptic defects induced by IBMPFD mutations. However, it does not suggest the irrelevance of dysregulation of protein degradation in IBMPFD. Perhaps after expression for six days in cultured neurons, protein degradation (through either proteasomal degradation or autophagy) is impaired and thus spine density is reduced, although the undegraded proteins have not yet formed aggregates. The involvement of ERAD, proteasomal degradation, and/or autophagy in VCP-dependent dendritic spine formation should be investigated directly in the future.

The previous studies had demonstrated that VCP forms complexes with cofactor p47 and regulates multiple membrane fusion events in cells [67,68], including nuclear envelope assembly [69], ER/Golgi membrane assembly [70,71], ER morphogenesis [72,73], and membrane fusion of autophagy [74]. Because p47 also coexists with VCP in the immunoprecipitated complex containing neurofibromin [8], it is likely that membrane fusion mediated by VCP also contributes to dendritic spine formation. More investigations have to been performed to address the possible role of VCP-mediated membrane fusion in dendritic spine formation.

### VCP and neuronal morphogenesis

Although the molecular mechanism underlying IBMPFD pathogenesis is still unclear, the accumulated evidence indicates that VCP plays critical roles in neuronal morphogenesis, which may account for the dementia phenotype associated with IBMPFD patients. Firstly, dystrophic neurites are frequently found in patients with FTD [55,75,76]. Moreover, VCP also regulates remodeling of dendritic arbor in Drosophila [77]. We also found that VCP is critical for the density of dendritic spines in cultured neurons. Expression of IBMPFD mutant VCP reduces the density of dendritic spines in cultured rat hippocampal neurons [8]. It is very possible that VCP actively contributes to neuronal morphogenesis and thus dysfunction of VCP may result in neurodegeneration.

### The interaction between neurofibromin and VCP

Using a variety of proteomic and biochemical methods, we identified a direct interaction between neurofibromin and VCP. The LRD of neurofibromin and the D1D2 region of VCP are required for the interaction [8]. We further showed that VCP acts downstream of neurofibromin in modulating dendritic spine formation. Compared with wild-type (WT) neurons, Nf1^+/- ^neurons have a lower density of dendritic spines [8]. Overexpression of WT VCP, but not the IBMPFD mutant, rescues the defects of spine density caused by Nf1 haploinsufficiency [8]. Moreover, the synaptic distribution of VCP is reduced in Nf1^+/- ^brains [8]. These results suggest that neurofibromin targets VCP to synapses and directs the function of VCP in spinogenesis.

To further elucidate the role of the neurofibromin-VCP interaction in NF1 and IBMPFD, the influence of mutations in the genes identified in patients has been investigated. The neurofibromin mutant (Y1587delta) lacking the Y1587 residue in the LRD has a much weaker interaction with VCP [8]. Patient carrying the Y1587delta mutation presents clinically with short stature and dementia [8], which echo the functions of VCP in dementia and bone metabolism. Moreover, unlike full length WT neurofibromin, expression of full length Y1587delta mutant does not rescue spine deficiency caused by Nf1 haploinsufficiency [8]. On the other hand, the VCP R155H and R95G mutations, the most common mutations in VCP, also reduce the interaction between VCP and neurofibromin [8]. In contrast to WT VCP, expression of these VCP mutants do not rescue the dendritic spine defects in Nf1^+/- ^neurons [8]. Taken together, these data show that reduction of the interaction between neurofibromin and VCP occurs in both NF1 and IBMPFD patients.

### Genetic modifications of NF1 and IBMPFD

Since the phenotypes of both NF1 and IBMPFD vary markedly, genetic modifications have been suggested to influence the function of neurofibromin and VCP. The interacting proteins of neurofibromin and VCP are possible genetic modifier candidates. Indeed, VCP interacts with different cofactors and regulates various cellular processes [43,59,64]. The variant expression levels of cofactors or other interacting proteins of VCP are likely to influence the functions of VCP. In addition to Ras and VCP, neurofibromin has been shown to also interact with syndecans [30,34], 14-3-3 [38], CRMP2/4 [35,39], and APP [37] (Figure 1). Our recent study indicated that overexpression of VCP rescues the defects of dendritic spines in cultured Nf1^+/- ^cortical neurons [8]. This suggests that variation of VCP protein levels affects the phenotypes caused by Nf1 haploinsufficiency. It is important to further explore this possibility in NF1 patients. Examination of the VCP expression levels and their correlation with the phenotypes of NF1 patients will provide clues whether the VCP expression levels influence the phenotypes of patients with NF1.

### The potential clinical effect of statin

Statin, an inhibitor of 3-hydroxy-3-methylglutaryl-coenzyme A (HMG-CoA) reductase, has been shown to rescue the learning and attention deficits of Nf1 mutant mice [78]. HMG-CoA reductase is the rate-limiting enzyme of the mevalonate pathway, which produces cholesterol and other isoprenoids. The role of statin in NF1 was examined because of the essential role of lipid modification in Ras activity, which is upregulated in NF1 mutant cells [79,80]. Additionally, cholesterol is involved in regulation of dendritic spinogenesis [81]. Thus, control of lipid metabolism may be critical for cognitive function. Recent studies indicated that VCP is required for downregulation of the protein levels of HMG-CoA reductase [82-84]. HMG-CoA reductase expression is tightly controlled by the concentration of sterol in cells, which influences both gene expression and protein stability of HMG-CoA reductase. VCP plays an essential role in sterol-induced dislocation of HMG-CoA reductase from the ER, which leads to ubiquitination and degradation of HMG-CoA reductase [82-84]. Thus, statin may correct the defects of Nf1 haploinsufficiency in both Ras- and VCP-dependent pathways. It also implies that statin may be applied to NF1 patients who have defects in the interaction between neurofibromin and VCP.

## Conclusions

The heterogeneity of patients' phenotypes presents an obstacle to establishing effective and specific clinical treatments for NF1 and IBMPFD. Understanding the molecular mechanisms of the pathogenesis of NF1 and IBMPFD, which still need more investigation to elucidate the finer details, is essential for overcoming these obstacles. Identification of the interacting molecules and elucidation of the crosstalk between or among the interacting proteins are certainly important to understanding the pathogenic mechanisms. The connection of the interaction between neurofibromin and VCP and the regulation of dendritic spinogenesis has provided the first molecular evidence that VCP functions as a genetic modifier for NF1. Studies in mouse genetic models and patients now have to be performed to confirm the role of VCP as a downstream effector of neurofibromin. It will also be interesting to explore whether mutations in the NF1 gene modulate the phenotypes of IBMPFD.

## Abbreviations

AAA ATPase: ATPases Associated with a variety of cellular Activities APTase; ALS: amyotrophic lateral sclerosis; CRMP: collapsing response mediator protein; CTD: C-terminal domain; Ena/VASP: Enabled/vasodilator-stimulated phosphoprotein; ERAD: endoplasmic reticulum-associated protein degradation; FTD: frontotemporal dementia; GRD: RasGAP-related domain; IBMPFD: Inclusion body myopathy with Paget disease of the bone and frontotemporal dementia; HMG-CoA: 3-hydroxy-3-methylglutaryl-coenzyme A; LRD: Leucine-rich domain; NF1: Neurofibromatosis Type I; TDP-43: TAR DNA binding protein 43; VCP: Valosin-containing protein.

## Competing interests

The author declares that they have no competing interests.

## Authors' contributions

Y-PH conceived and prepared this review.
